# Supplementary material for: ChromBERT: A foundation model for learning interpretable representations for context-specific transcriptional regulatory networks
Source: Cell Genom. 2026 Jan 26;6(4):101130. doi: 10.1016/j.xgen.2025.101130 (PMC13069865; doi:10.1016/j.xgen.2025.101130)
Supplement: Document S1. Figures S1–S11 [file mmc1.pdf]

Cell Genomics, Volume *6*

Supplemental information

ChromBERT: A foundation model for learning interpretable representations for context-specific transcriptional regulatory networks

Zhaowei Yu, Dongxu Yang, Qianqian Chen, Yuxuan Zhang, Zhanhao Li, Yucheng Wang, Chenfei Wang, and Yong Zhang

Supplemental Figures

Figure S1

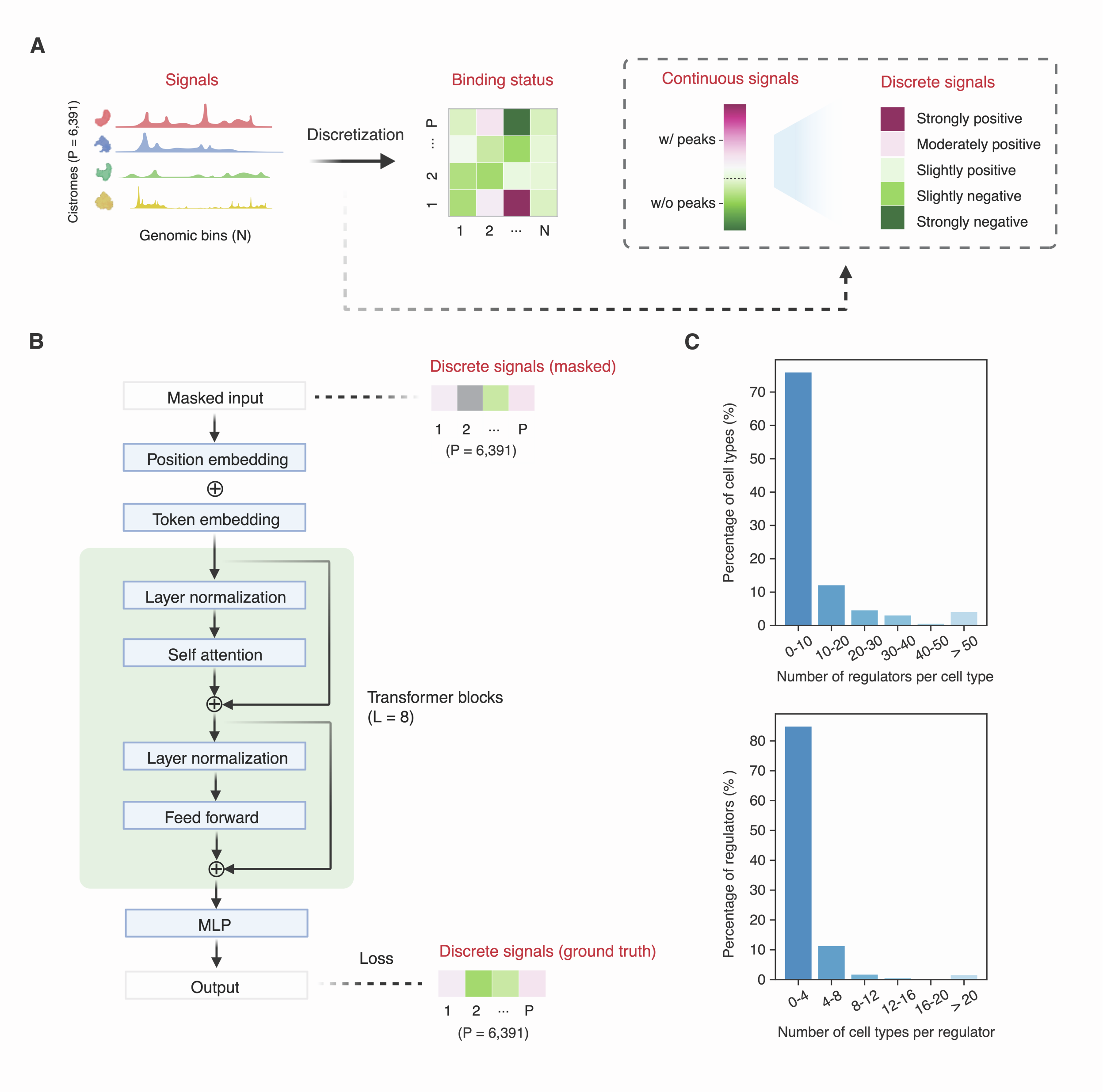

Figure S1. Pre-training dataset and workflow, related to Figure 1 and STAR Methods.

(A) Schematic illustrating the tokenization process of the Cistrome-Human-6K datasets. Continuous signals for each cistrome were converted into discrete signals and categorized into five categories from low to high signals (see STAR Methods).

(B) Schematic showing pre-trained model architecture of ChromBERT, which includes two embedding layers to handle token (binding status) and positional information (the identity of cistrome), eight transformer blocks designed to capture co-association pattern among cistromes, and an MLP decoder to predict the binding status of cistromes. The model parameters were updated effectively through loss calculations on masked objectives (see STAR Methods).

(C) Bar plots showing the data distribution of cell types and regulators in the Cistrome-Human-6K dataset. The majority of cell types have available cistromes for fewer than 10 regulators (top), while most regulators have cistromes present in fewer than 4 cell types (bottom), highlighting the sparsity of the dataset's coverage.

Figure S2

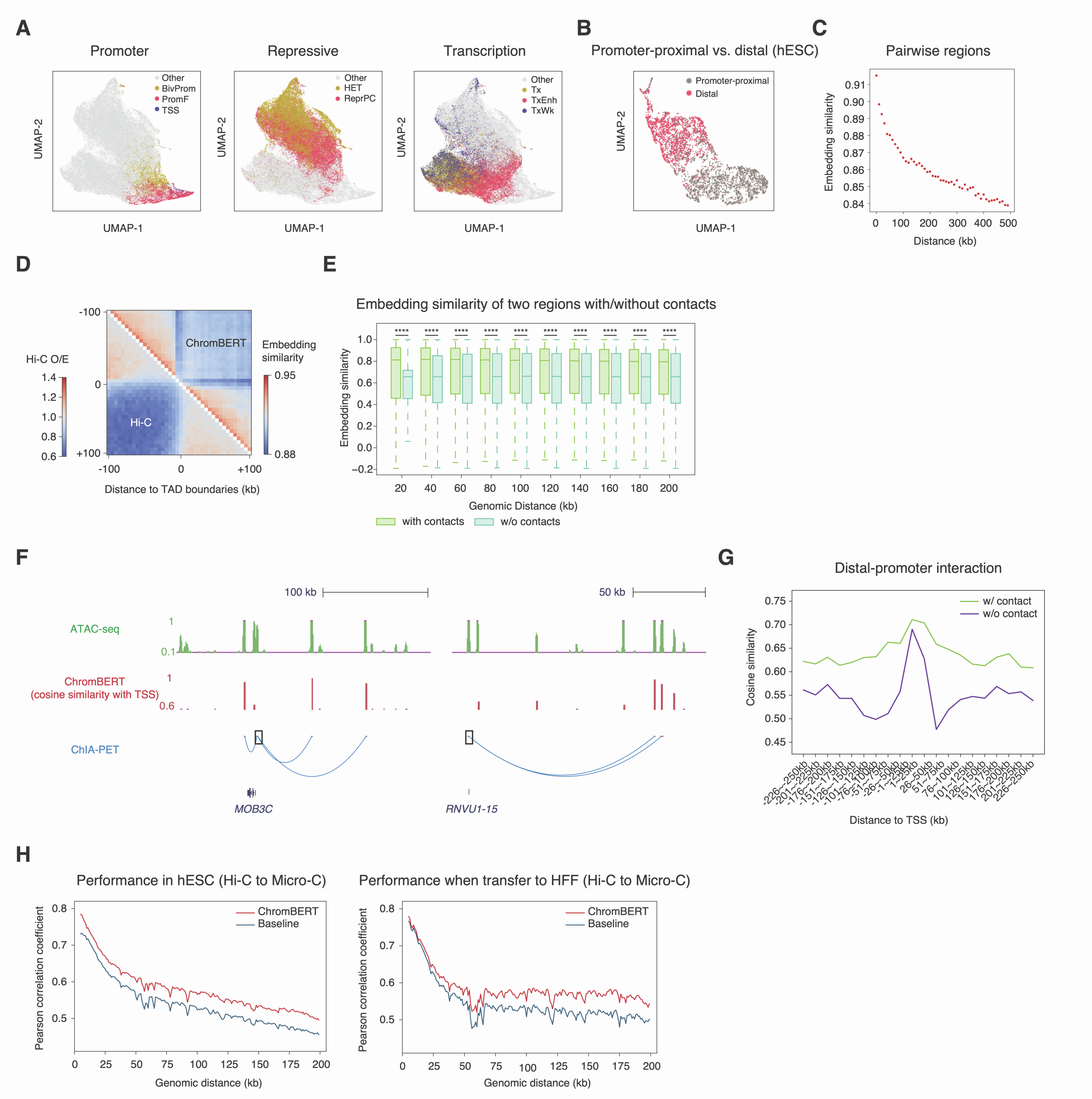

Figure S2. Pre-trained TRN embeddings represent human TRNs, related to Figure 1.

(A) Uniform Manifold Approximation and Projection (UMAP)[1] visualization of ChromBERT's region-level TRN embedding vectors across regions on chromosome one, color-coded by chromatin states as reported in the previous publication[2]. For each region, the region-level TRN embedding vector was obtained by averaging TRN embedding matrix (number of regulators × hidden dimension) across regulator dimension within that region.

(B) UMAP visualization of region-level TRN embedding vectors from promoter-proximal and distal regions in hESCs. Each point represents embedding of a genomic region on chromosome 1. Promoter-proximal regions were defined as ±500 bp around accessible TSSs, while distal regions were defined as accessible regions located more than 10 kb away from the nearest TSS. Chromatin accessibility was determined using ATAC-seq data from hESCs (GEO accession: GSM2386582[3]).

(C) Averaged pairwise cosine similarity of region-level TRN embedding vectors from ChromBERT across 761,838 randomly selected genomic bin pairs on chromosome 1. Cosine similarity between pairs of these region-level averaged TRN embedding vectors was calculated as a function of genomic distance. Specifically, all region pairs separated by a given genomic distance were grouped into bins, and the average cosine similarity across all pairs within each bin was reported.

(D) Bottom triangle: heatmap showing aggregated Hi-C interactions, indicated by observed/expected (O/E) values, centered around TAD boundaries on chromosome one. Top triangle: heatmap illustrating the pairwise cosine similarity of region-level TRN embedding vectors for genomic bin pairs. The used Hi-C data in K562 cells was from the previous study (GSE63525[4]).

(E) Box plots showing the cosine similarity of ChromBERT's region-level TRN embedding vectors for pairs of genomic regions in hESC cells, stratified by the presence (green) or absence (blue) of Micro-C contacts across different genomic distances. Region pairs with Micro-C contacts consistently exhibit significantly higher cosine similarity than those without at all distances. Statistical significance was assessed using a two-sided Student's *t*-test, with **** indicating *p*-value < 1×10^{-4}. The center lines mark the median, the box limits indicate the 25th and 75th percentiles, and the whiskers extend to 1.5× the interquartile range from the 25th and 75th percentiles. Each distance group has at least 728,667 region pairs.

(F) UCSC Genome Browser views showing representative enhancer–promoter contacts for *MOB3C* (chr1:46,539,804-46,780,591; left) and *RNVU1-15* (chr1:144,400,267-144,583,599; right) and their embedding similarities. Tracks from top to bottom: ATAC-seq signal in hESCs (GSM2386582[3]; green), cosine similarity between ChromBERT's region-level TRN embedding vectors for TSS-located 1-kb bin (boxed) and nearby accessible distal regions (red), and chromatin loops detected by Pol II ChIA-PET in hESCs (ENCSR782EKZ; blue arcs). Only loops linking accessible distal regions to the target promoters are shown.

(G) Line plots showing cosine similarity of region-level TRN embedding vectors between distal-promoter region pairs. Dark green and light purple lines indicate distal-promoter pairs with and without chromatin contacts, respectively, as determined by Micro-C data in hESCs.

(H) Line plot illustrating the distance-stratified Pearson correlation coefficient between observed Micro-C contact maps and CNN-predicted contact maps derived from ChromBERT's TRN embeddings and Hi-C contact maps in hESC cells (see STAR Methods). The baseline comparison is made using Hi-C contact maps interpolated from 5-kb to 1-kb resolution. The CNN model was trained and tested in hESC cells (left) and directly transferred to HFF cells (right). The Micro-C and Hi-C data used in hESC cells (4DNES21D8SP8, 4DNES2M5JIGV) and HFF cells (4DNESWST3UBH, 4DNES2R6PUEK) were obtained from previous study[5].

Figure S3

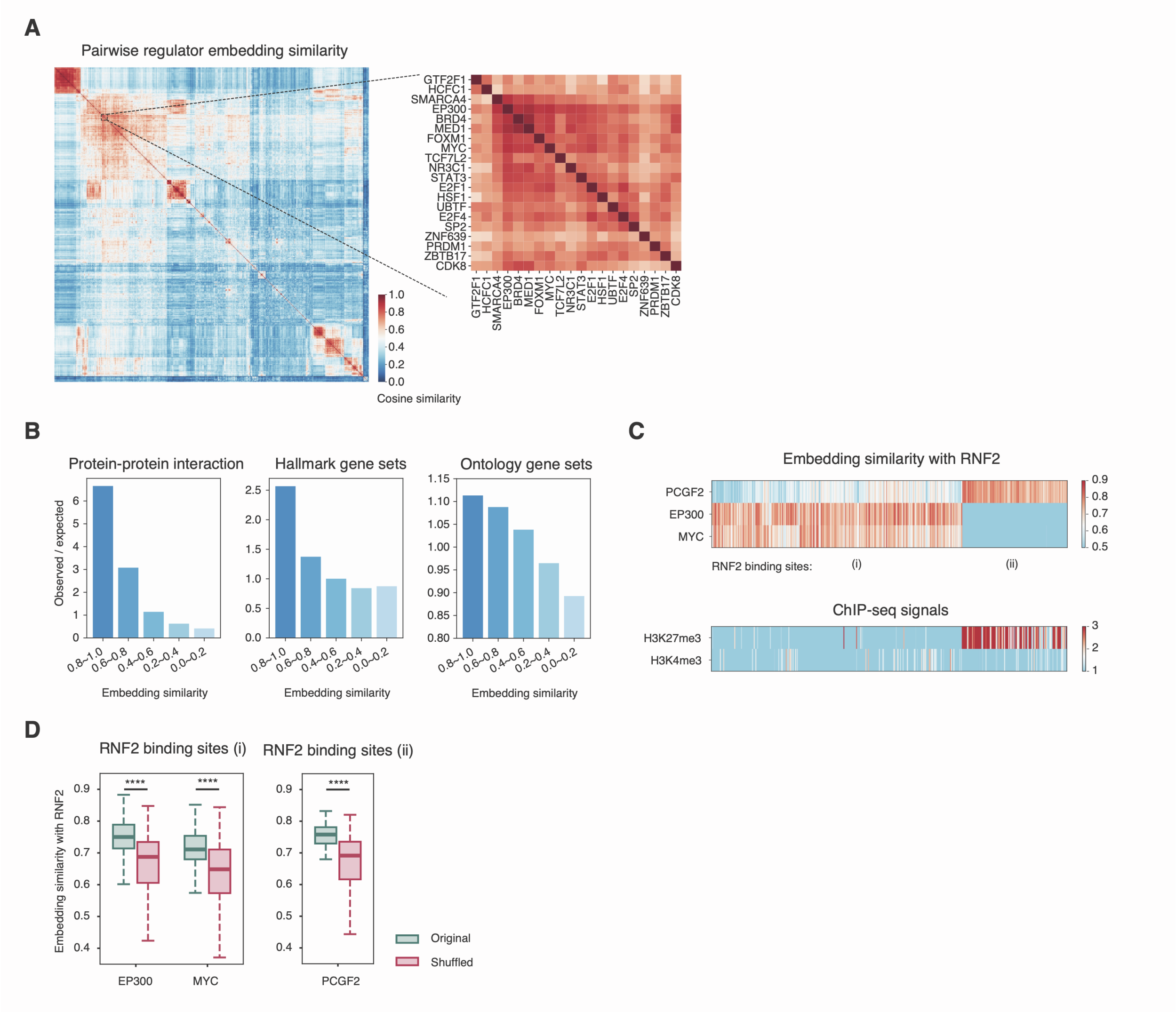

Figure S3. Pre-trained regulator embeddings represent human TRNs, related to Figure 1.

(A) Heatmap depicting the cosine similarities of ChromBERT's regulator embeddings on chromosome one, revealing complex interaction between transcription regulators (left). A zoomed-in view highlights that functionally associated regulators, such as EP300 and BRD4, have higher embedding similarities (right). For each regulator, regulator embeddings were compiled from regions across chromosome one into a 768-dimensional vector, facilitating the comprehensive analysis.

(B) Bar plot shows pre-trained ChromBERT's regulator embeddings can represent the functional collaborations among transcription regulators. Regulator pairs were ranked by the cosine similarity between their averaged embeddings across chromosome one. The observed (each group)/expected (all pairs) ratios for protein-protein interaction frequency (BioPlex[6]), co-occurrence frequency in the same hallmark gene sets or ontology gene sets (Molecular Signatures Database[7]) were shown, highlighting significant functional collaborations among regulators with high embedding similarity.

(C) Top, heatmap shows the embedding similarities between RNF2 and other regulators at representative RNF2 peaks. The color represents the cosine similarity of two regulator embeddings. Bottom, the H3K4me3 and H3K27me3 ChIP-seq profiles at the given genomic regions. The color represents ChIP-seq signals in

RPM. RNF2, H3K4me3 and H3K27me3 ChIP-seq data were from previous study (GSE105028[8], GSE39912[9] and GSE62562[10]).

(D) Boxplots showing cosine similarity between RNF2 embeddings and those of other regulators under original (light green) or shuffled (pink) conditions within subset of RNF2 binding sites. Shuffling was performed simultaneously for PCGF2 ($n = 124$), EP300 and MYC ($n = 296$) across the full set of RNF2's representative peaks. This approach preserved their overall occurrence frequencies and pairwise co-occurrence rates with RNF2. Statistical significance was assessed using a two-sided Student's *t*-test, with **** indicating *p*-value < 1×10^{-4}. The center lines mark the median, the box limits indicate the 25th and 75th percentiles, and the whiskers extend to 1.5× the interquartile range from the 25th and 75th percentiles.

Figure S4

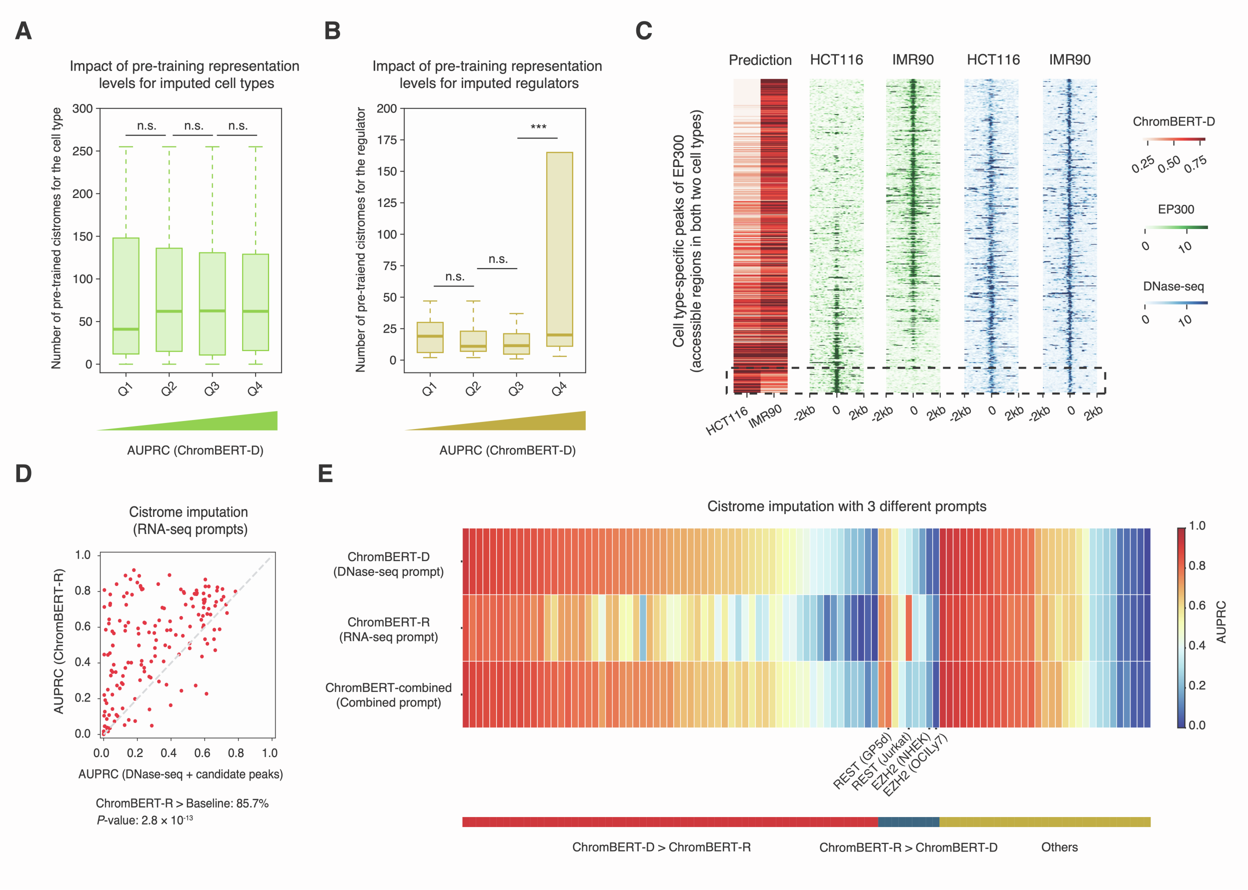

Figure S4. Robustness of ChromBERT in cistrome imputation, related to Figure 2.

(A and B) The impact of pre-training representation levels on the performance of cistrome imputation via ChromBERT-D. The *X*-axis represents performance quantiles on cistrome imputation, divided into four groups: the first quantile (Q1, 0–25%, $n = 61$), second quantile (Q2, 25–50%, $n = 60$), third quantile (Q3, 50–75%, $n = 60$), and fourth quantile (Q4, 75–100%, $n = 61$), ordered from low to high performance. The *Y*-axis indicates the pre-training representation levels (number of cistromes) for the cell type (A) or the transcription regulator (B) of the test cistrome being imputed. Statistical significance was conducted using a two-sided Student's *t*-test, where n.s. indicates non-significant and *** indicates *p*-value < 0.001. The center lines mark the median, the box limits indicate the 25th and 75th percentiles, and the whiskers extend to 1.5× the interquartile range from the 25th and 75th percentiles.

(C) Heatmaps present ChromBERT-D's predicted probabilities using DNase-seq prompts (red), alongside ChIP-seq signals (green) and DNase-seq signals (blue) on cell-type-specific peaks of EP300 that were accessible in both HCT116 and IMR90 (see STAR Methods). The EP300 ChIP-seq and DNase-seq signals were normalized to the genome average. Black dashed rectangles highlight cell-type-specific binding events that do not differ in DNase-seq signals, yet were accurately predicted by ChromBERT-D.

(D) Scatter plot illustrating the performance of cistrome imputation tasks for ChromBERT-R using bulk-cell RNA-seq prompts ($n = 168$ cistromes), compared to baseline methods measured by DNase-seq signal and candidate peaks (see STAR Methods). The diagonal gray line indicates equal performance, with percentages of cistromes where ChromBERT-R outperforms annotated alongside the *p*-value calculated by a two-sided Student's *t*-test.

(E) Benchmarking performance for three cell-type-specific prompts for cistrome imputation. Heatmap showing the AUPRC for 101 test cistromes using three prompt configurations: DNase-seq prompts

(ChromBERT-D), RNA-seq prompts (ChromBERT-R), and combined prompts (concatenation of DNase-seq prompts and RNA-seq prompts; ChromBERT-combined). Cistromes were grouped into three clusters: ChromBERT-D outperformed ChromBERT-R by AUPRC > 0.05, ChromBERT-R outperformed ChromBERT-D by AUPRC > 0.05, and others.

Figure S5

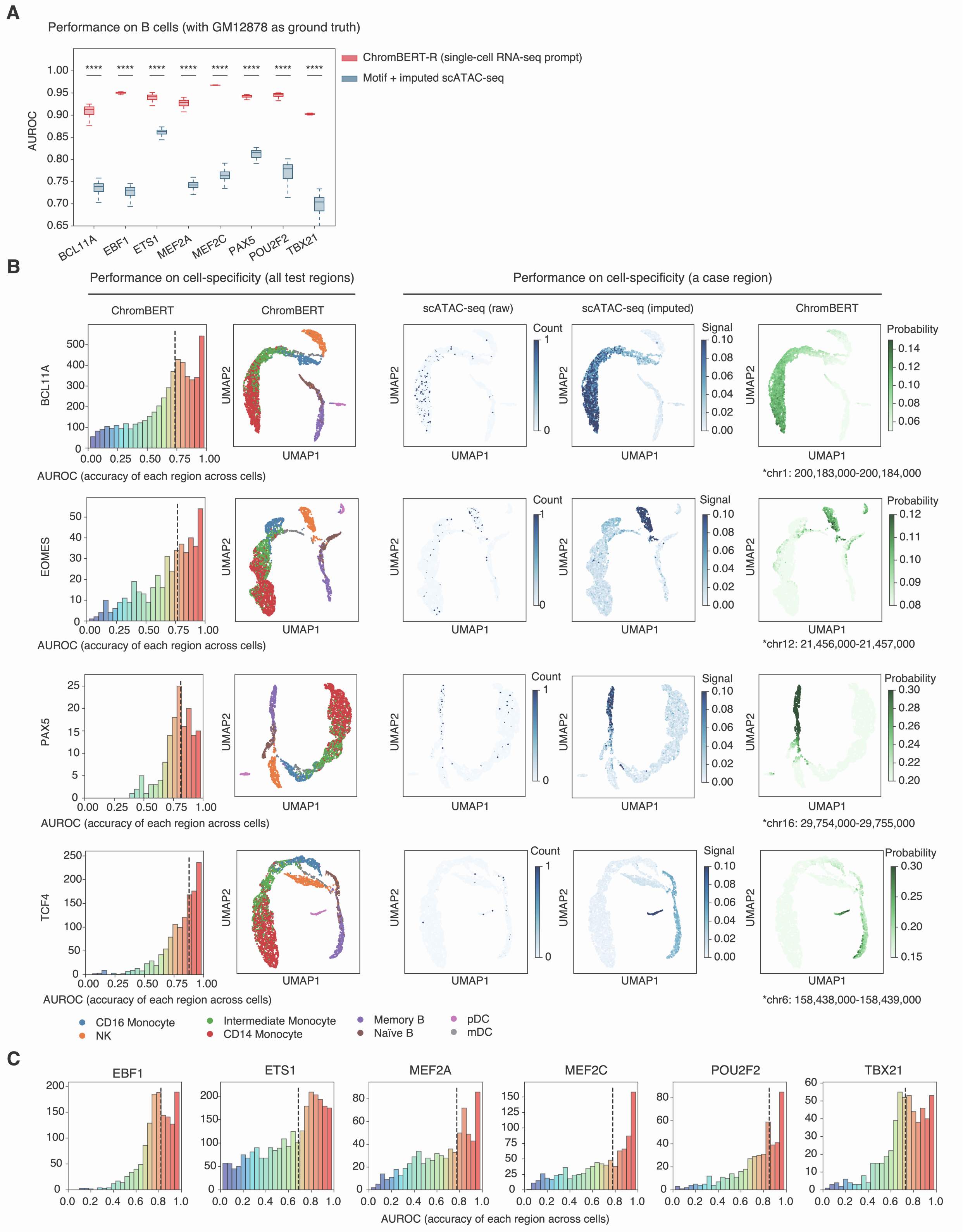
A
Performance on B cells (with GM12878 as ground truth)
ChromBERT-R (single-cell RNA-seq prompt)
Motif + imputed scATAC-seq
AUROC
BCL11A
EBF1
ETS1
MEF2A
MEF2C
PAX5
POU2F2
TBX21
B
Performance on cell-specificity (all test regions)
Performance on cell-specificity (a case region)
ChromBERT
scATAC-seq (raw)
scATAC-seq (imputed)
Count
Signal
Probability
UMAP1
UMAP2
BCL11A
EOMES
PAX5
TCF4
AUROC (accuracy of each region across cells)
*chr1: 200,183,000-200,184,000
*chr12: 21,456,000-21,457,000
*chr16: 29,754,000-29,755,000
*chr6: 158,438,000-158,439,000
CD16 Monocyte
NK
Intermediate Monocyte
CD14 Monocyte
Memory B
Naïve B
pDC
mDC
C
EBF1
ETS1
MEF2A
MEF2C
POU2F2
TBX21
AUROC (accuracy of each region across cells)

Figure S5. ChromBERT imputes cistromes at single-cell resolution using scGPT prompts, related to Figure 2.

(A) Box plots comparing prediction performance in B cells using bulk-cell ChIP-seq data from GM12878 as the ground truth. Red boxes represent ChromBERT with single-cell prompts, while blue boxes indicate a combination of DNA-binding motifs and imputed single-cell ATAC-seq signals (via scOpen[11]). The comparison was performed at 1-kb bins containing imputed single-cell ATAC-seq peaks (imputed single-cell ATAC-seq signals > 0.1) in the PBMC dataset and the presence of motifs for each regulator. Statistical significance was computed with the two-sided Student's *t*-test, where **** represents *p*-value < 1×10^{-4}. The center lines mark the median, the box limits indicate the 25th and 75th percentiles, and the whiskers extend to 1.5× the interquartile range from the 25th and 75th percentiles. Each group has $n = 1{,}920$ cells.

(B) Cell-specificity analysis of cistrome imputation by ChromBERT with single-cell prompts. The first and second columns evaluate performance across all test regions: the first column shows the distribution of AUROC scores for cell-specificities of each region, and the second column presents a UMAP analysis of predictive probabilities across cells, with cells colored by pseudo-bulk cell type annotations (from paired single-cell RNA-seq). The third to fifth columns focus on the cell-specificity of a case region: the third column shows raw ATAC-seq counts, the fourth displays imputed ATAC-seq counts, and the fifth presents ChromBERT's predictions across different cells. Test regions were defined as a merge set of regions showing high chromatin accessibility specificity in each pseudo-bulk cell type. Each single cell was assigned to a pseudo-bulk cell type based on single-cell RNA-seq annotations. Specific regions for each pseudo-bulk cell type were defined as those accessible (with imputed single-cell ATAC-seq peak) in at least 60% of cells assigned to that cell type and accessible in no more than 10% of other cells, with a mean fold change in accessible cell number between the two groups of cells > 2. All pseudo-bulk cell-type-specific regions were pooled as total test regions for this analysis, while evaluation for each specific regulator was only performed in total regions with the regulator's motif. Pseudo-bulk cell types related to T cells are excluded due to insufficient representation by scGPT.

(C) Cell-specificity analysis of cistrome imputation by ChromBERT with single-cell prompts for additional transcription regulators.

Figure S6

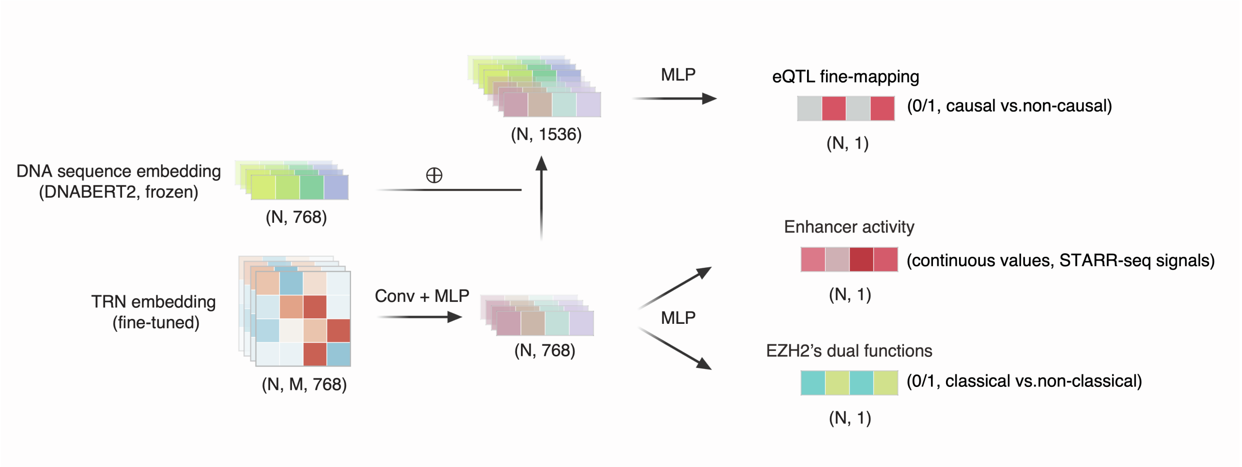

Figure S6. Schematic illustration of the workflow for the three fine-tuning downstream tasks, related to Figure 3 and STAR Methods.

Figure S7

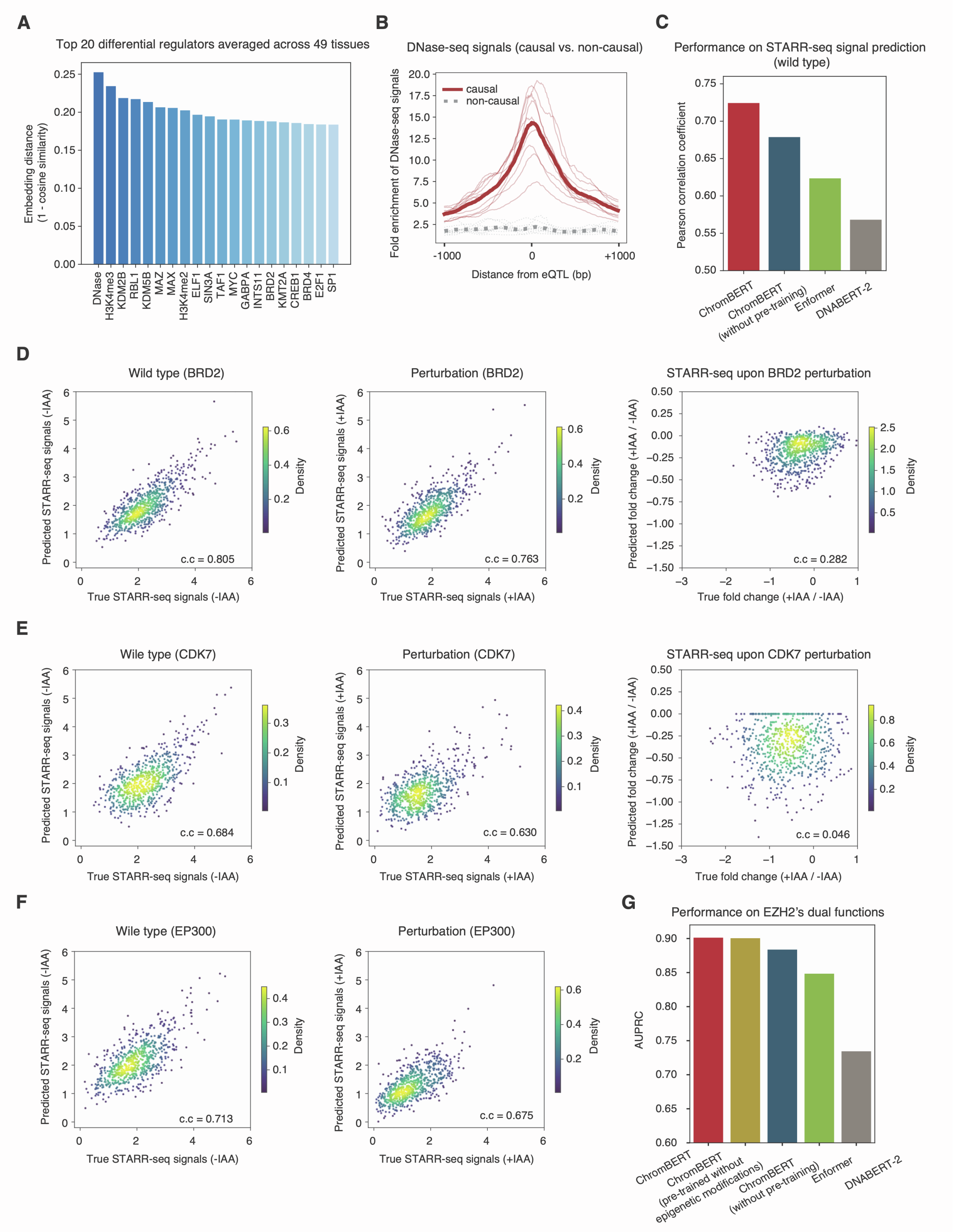

Figure S7. Context-specificities of TRNs revealed by ChromBERT, related to Figure 3.

(A) Bar plot illustrating the top 20 transcription regulators ranked by embedding shift between causal and non-causal eQTLs, averaged across 49 tissues.

(B) Average DNase-seq site profiles around causal versus non-causal eQTLs. Each thin line represents an individual tissue (10 tissues used, see Data Availability for details), while the bold line represents the mean profiles across 10 tissues. Red lines indicate causal eQTLs and gray dashed lines indicate non-causal eQTLs.

(C) Bar plots illustrate the performance of ChromBERT, ChromBERT (without pre-training), Enformer, and DNABERT-2 in modeling STARR-seq signals in wild type HCT116 cells.

(D-F) Scatter plots showing the prediction performance of ChromBERT for in silico perturbation studies. The predicted and ground truth wild type STARR-seq signals (left), IAA-treated (perturbation of factors) STARR-seq signals (center) and $\log_2$-transformed fold change of perturbation versus wild-type (right) were shown, respectively. Analyses were conducted using STARR-seq datasets with perturbation for three cofactors, BRD2 (D), CDK7 (E), and EP300/CREBBP (F) (GSE156740[12]). Pearson correlation coefficients were annotated in the plot, and the color represents the density of points.

(G) Performance comparisons of different models on EZH2's dual function classification. Bar plots show the performance of ChromBERT, ChromBERT (pre-trained without epigenetic modifications), ChromBERT (without pre-training), Enformer, and DNABERT-2 in classifying EZH2 binding sites as classical (H3K27me3-associated) or non-classical (H3K27me3-independent). ChromBERT was fine-tuned by omitting cistromes associated with H3K27me3 from input reference cistromes to diminish its dominant role in the task. ChromBERT (pre-trained without epigenetic modifications) is a pre-trained version of ChromBERT pre-trained only on transcription regulator cistromes, excluding all epigenetic modification tracks including H3K27me3, to eliminate any potential advantage from prior exposure to these signals. EZH2 and H3K27me3 ChIP-seq data were from previous studies (GSE61176 and GSE29611[13]).

Figure S8

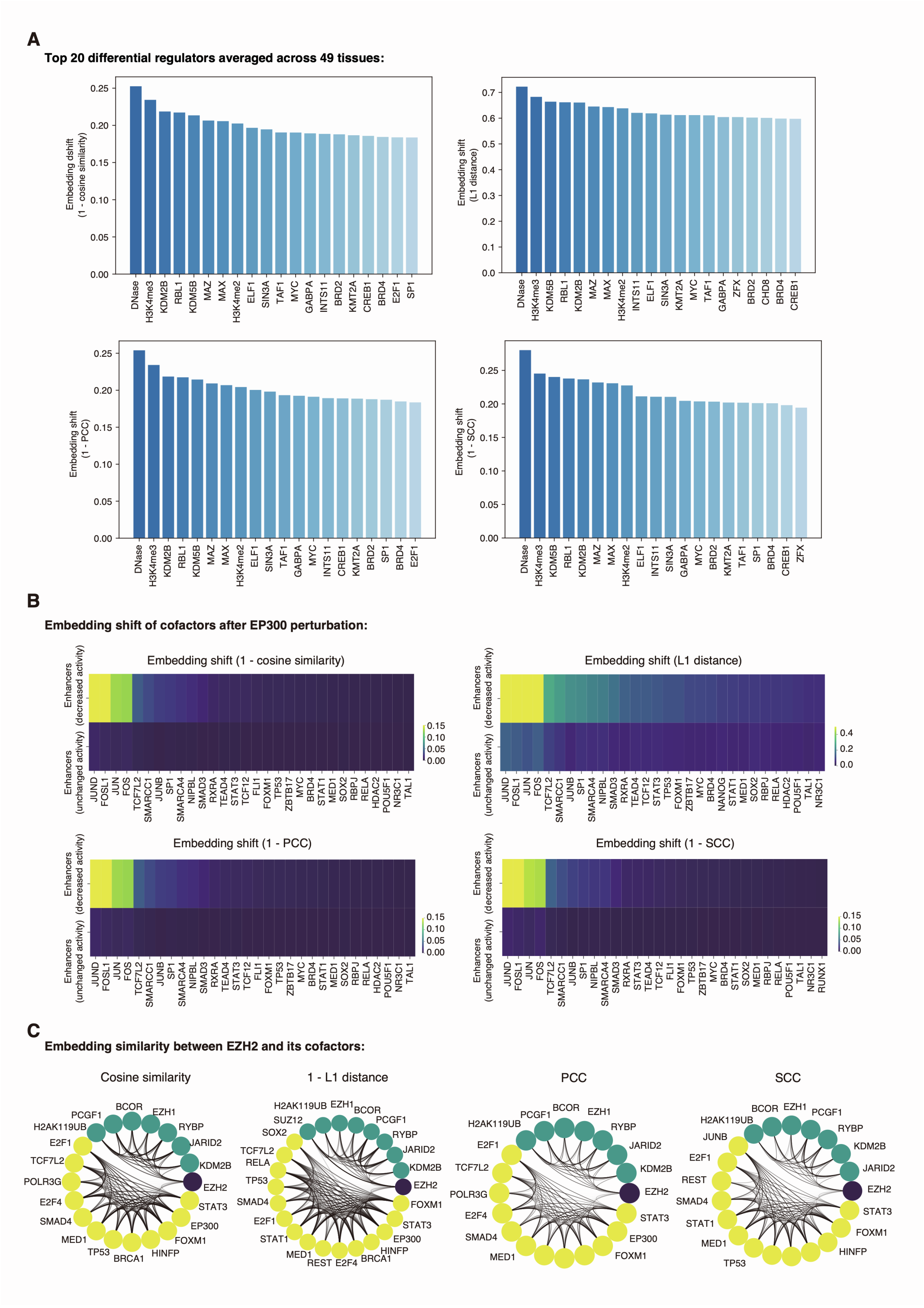
A
Top 20 differential regulators averaged across 49 tissues:
Embedding dshift (1 - cosine similarity)
Embedding shift (L1 distance)
Embedding shift (1 - PCC)
Embedding shift (1 - SCC)
B
Embedding shift of cofactors after EP300 perturbation:
Embedding shift (1 - cosine similarity)
Embedding shift (L1 distance)
Embedding shift (1 - PCC)
Embedding shift (1 - SCC)
Enhancers (decreased activity)
Enhancers (unchanged activity)
C
Embedding similarity between EZH2 and its cofactors:
Cosine similarity
1 - L1 distance
PCC
SCC

Figure S8. Interpretation analyses across three downstream tasks using different similarity or distance metrics, related to Figure 3.

Four metrics for quantifying embedding relationships: cosine similarity, L1-normalized L1 distance, Pearson correlation coefficient (PCC), and Spearman correlation coefficient (SCC).

(A) Bar plot illustrating the top 20 transcription regulators ranked by embedding shift between causal and non-causal eQTLs, averaged across 49 tissues. Panels (left to right, top to bottom) show four embedding shift measures: 1-cosine similarity, L1 distance, 1-PCC and 1-SCC.

(B) Heatmap showing embedding shift for potential cofactors of EP300 at enhancers before and after the perturbation of EP300/CREBBP. Two enhancer groups are shown: activity-decreased enhancers ($\log_2$ fold change < -1, $n = 164$) and activity-unchanged enhancers (-0.5 < $\log_2$ fold change < 0.5, $n = 343$). Panels show four embedding shift measures.

(C) Circos plot showing embedding similarities between EZH2 and other regulators at classical (with H3K27me3) and non-classical (without H3K27me3) genomic loci. Two groups of regulators are highlighted: (i) classical group (green) shows higher embedding similarity with EZH2 at classical loci compared to non-classical loci (embedding similarity with EZH2 at classical loci ranking the top 5% among all regulators and the embedding similarity difference between classical loci and non-classical loci > 0.1); (ii) the non-classical group (yellow) exhibits the converse pattern. Each node represents a regulator, and the transparency of edges linking two nodes represent the embedding similarity of two regulators, only edges with high pairwise embedding similarity (> 0.8) were plotted. Panels show four embedding similarity measures.

Figure S9

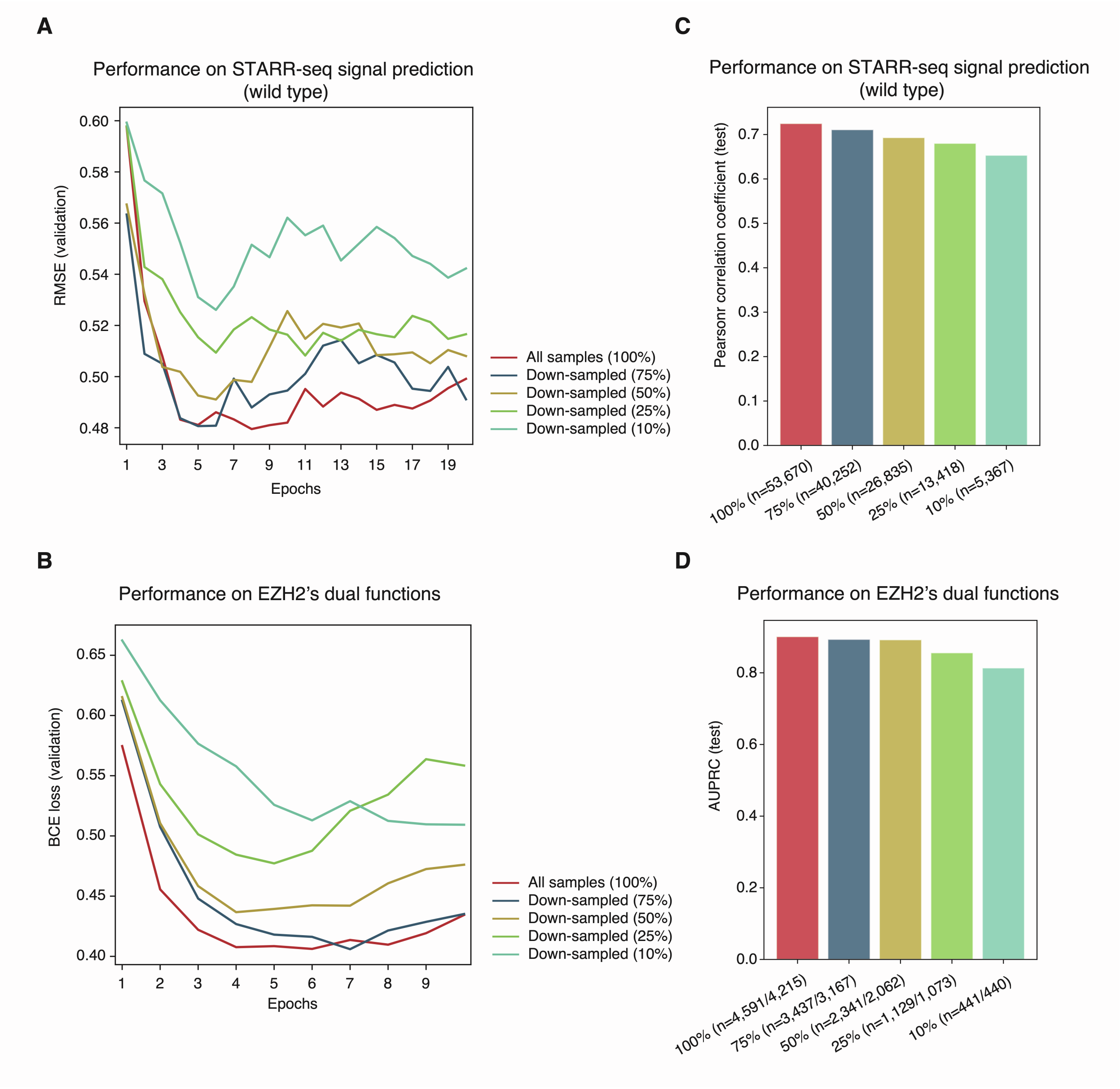

Figure S9. Performance of ChromBERT across two downstream tasks using the full training dataset (100%) and down-sampled subsets (75%, 50%, 25%, and 10%), related to Figure 3.

(A) Line plots show the validation RMSE over training epochs for ChromBERT trained on different down-sampled training sets for the task of modeling STARR-seq signals in wild-type HCT116 cells.

(B) Line plots show the validation BCE loss over training epochs for ChromBERT trained on different down-sampled training sets for classifying EZH2's classical and non-classical sites.

(C) Bar plots show ChromBERT's performance across down-sampled training sets for modeling STARR-seq signal in wild-type HCT116 cells. The notation $(n = x)$ indicates the total number of samples in the training dataset.

(D) Bar plots show ChromBERT's performance across down-sampled training sets for classifying EZH2 classical vs non-classical sites. The notation $(n = x/y)$ indicates the number of positive and negative samples in the training dataset.

Figure S10

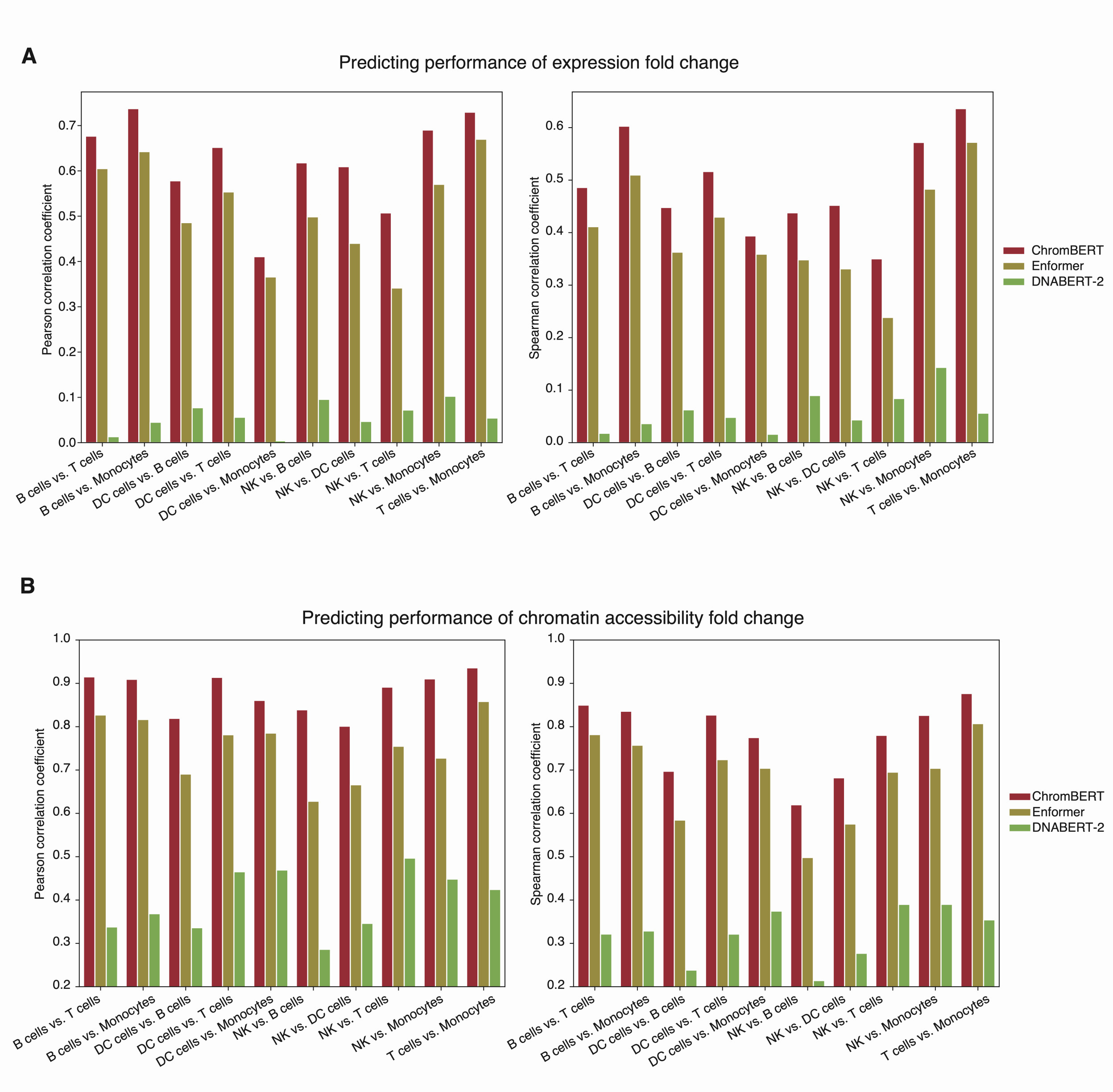

Figure S10. Benchmark on prediction for transcriptome and chromatin accessibility changes between single-cell pseudo-bulk cell types, related to Figure 4.

(A and B) Bar plots comparing performances of ChromBERT, Enformer and DNABERT-2 in predictions for genome-wide transcriptome (A) and chromatin accessibility (B) changes between different single-cell pseudo-bulk cell types. Pearson correlation coefficient and Spearman correlation coefficient were computed and shown.

Figure S11

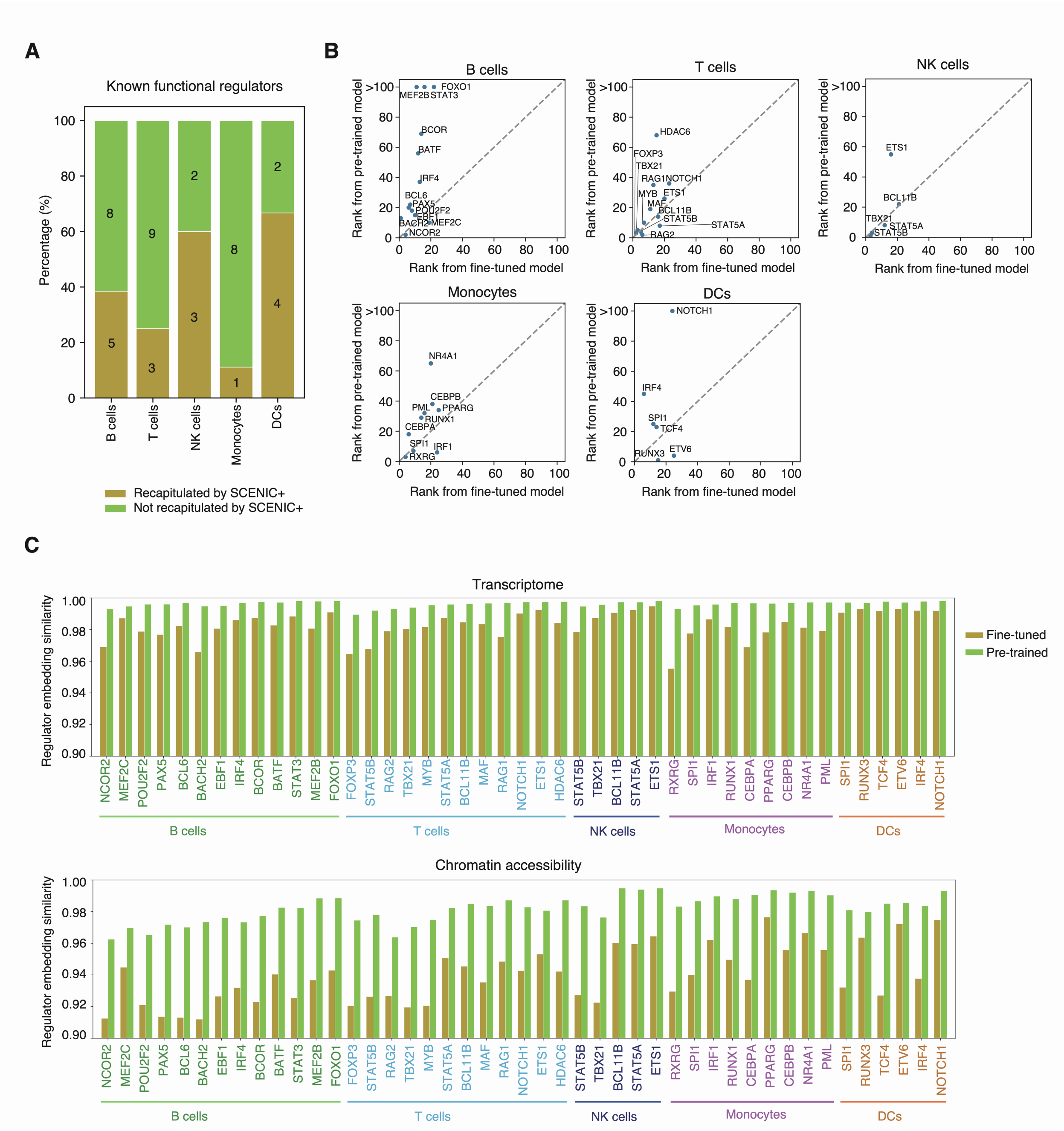

Figure S11. Identified key regulators for cellular heterogeneity, related to Figure 4.

(A) Bar plots illustrating the percentage of known key regulators identified by ChromBERT in various cell types that were not detected by SCENIC+ (v1.0.1)[14].

(B) Scatter plots comparing the rankings of known key regulators using fine-tuned regulator embeddings (*X*-axis) versus pre-trained regulator embeddings (*Y*-axis).

(C) Bar plots showing the comparison of embedding similarity for known key regulators between regions with increased activity and unchanged activity. The plots reveal that known key regulators exhibit lower

embedding similarity with fine-tuned regulator embeddings compared to pre-trained regulator embeddings, suggesting that fine-tune model can capture important regulator in determining cellular heterogeneity.

Figure S12

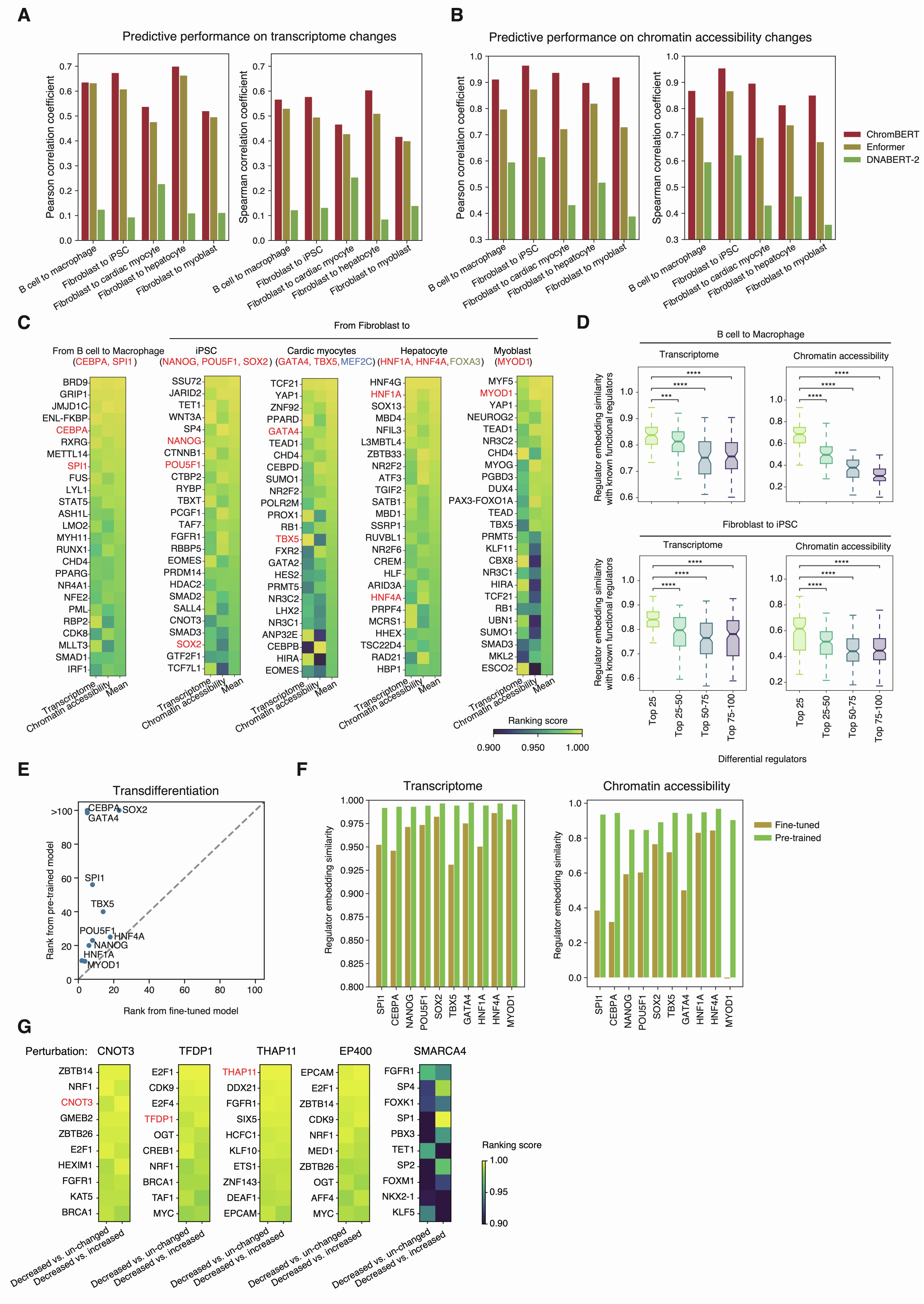
A
Predictive performance on transcriptome changes
Pearson correlation coefficient
Spearman correlation coefficient
B cell to macrophage
Fibroblast to iPSC
Fibroblast to cardiac myocyte
Fibroblast to hepatocyte
Fibroblast to myoblast
B
Predictive performance on chromatin accessibility changes
ChromBERT
Enformer
DNABERT-2
C
From Fibroblast to
From B cell to Macrophage
(CEBPA, SPI1)
iPSC
(NANOG, POU5F1, SOX2)
Cardic myocytes
(GATA4, TBX5, MEF2C)
Hepatocyte
(HNF1A, HNF4A, FOXA3)
Myoblast
(MYOD1)
Transcriptome
Chromatin accessibility
Mean
Ranking score
D
B cell to Macrophage
Transcriptome
Chromatin accessibility
Fibroblast to iPSC
Regulator embedding similarity with known functional regulators
Top 25
Top 25-50
Top 50-75
Top 75-100
Differential regulators
E
Transdifferentiation
Rank from pre-trained model
Rank from fine-tuned model
F
Transcriptome
Chromatin accessibility
Regulator embedding similarity
Fine-tuned
Pre-trained
G
Perturbation: CNOT3
TFDP1
THAP11
EP400
SMARCA4
Decreased vs. un-changed
Decreased vs. increased
Ranking score

Figure S12. Identified key regulators for transdifferentiation, related to Figure 4.

(A and B) Bar plots comparing performances of ChromBERT, Enformer and DNABERT-2 in predictions for genome-wide transcriptome (A) and chromatin accessibility (B) changes between different cell states. Pearson correlation coefficient and Spearman's correlation coefficient were computed and shown.

(C) Heatmap shows ranking scores of the top 25 regulators identified by ChromBERT. The known driver regulators of each transdifferentiation process were annotated above the heatmaps and known drivers successfully recapitulated by ChromBERT were highlighted in red. MEF2C, not recapitulated by ChromBERT, was marked in blue. And FOXA3, lacking related cistromes in our model, was marked in brown.

(D) Box plots showing embedding similarity between top-ranked regulators (w/o known driver regulators) and known driver regulators, suggesting the top-ranked regulators have high potential function associations with known driver regulators. Significance tests were performed by using two-sided Student's *t*-tests. *** represents *p*-value < 0.001, **** represents *p*-value < 1×10^{-4} and ns represents non-significant. For the B cell to macrophage transition, each group has at least 72 regulator-known pairs. For the fibroblast to iPSC transition, each group has at least 140 regulator-known driver pairs.

(E) Scatter plots comparing the ranking of known driver regulators by interpreting fine-tuned regulator embeddings (*X*-axis) or pretrained regulator embeddings (*Y*-axis) (see STAR Methods).

(F) Bar plots comparing the embedding similarity of known driver regulators between regions with increased activity and unchanged activity, highlighting that known driver regulators show lower embedding similarity with fine-tuned regulator embeddings than pre-trained regulator embeddings. It suggests that fine-tuned model can capture important regulators in determining cellular state transition more effectively.

(G) Validation of embedding shift-based interpretation using regulator perturbation ATAC-seq datasets. Heatmap showing the top 10 transcription regulators exhibiting the largest embedding shifts between decreased and un-changed or increased regions following perturbation. The perturbed regulators are highlighted in red. Chromatin accessibility changes were analyzed using ATAC-seq datasets in eHAP cell line (GSE144448[15]), including control and knockout samples for CNOT3, EP400, TFDP1, THAP11, and SMARCA4. The top 200 regions showing the greatest decrease or increase in ATAC-seq signal, together with 2,000 un-changed regions, were used for embedding-shift analysis to identify key regulators, following a strategy similar to that used for driver regulator identification during transdifferentiation.

Supplemental References

1. Becht, E., McInnes, L., Healy, J., Dutertre, C.A., Kwok, I.W.H., Ng, L.G., Ginhoux, F., and Newell, E.W. (2018). Dimensionality reduction for visualizing single-cell data using UMAP. Nat Biotechnol. 10.1038/nbt.4314.
2. Vu, H., and Ernst, J. (2022). Universal annotation of the human genome through integration of over a thousand epigenomic datasets. Genome Biol *23*, 9. 10.1186/s13059-021-02572-z.
3. Diao, Y., Fang, R., Li, B., Meng, Z., Yu, J., Qiu, Y., Lin, K.C., Huang, H., Liu, T., Marina, R.J., et al. (2017). A tiling-deletion-based genetic screen for cis-regulatory element identification in mammalian cells. Nat Methods *14*, 629-635. 10.1038/nmeth.4264.
4. Rao, S.S., Huntley, M.H., Durand, N.C., Stamenova, E.K., Bochkov, I.D., Robinson, J.T., Sanborn, A.L., Machol, I., Omer, A.D., Lander, E.S., and Aiden, E.L. (2014). A 3D map of the human genome at kilobase resolution reveals principles of chromatin looping. Cell *159*, 1665-1680. 10.1016/j.cell.2014.11.021.
5. Krietenstein, N., Abraham, S., Venev, S.V., Abdennur, N., Gibcus, J., Hsieh, T.S., Parsi, K.M., Yang, L., Maehr, R., Mirny, L.A., et al. (2020). Ultrastructural Details of Mammalian Chromosome Architecture. Mol Cell *78*, 554-565.e557. 10.1016/j.molcel.2020.03.003.
6. Huttlin, E.L., Bruckner, R.J., Navarrete-Perea, J., Cannon, J.R., Baltier, K., Gebreab, F., Gygi, M.P., Thornock, A., Zarraga, G., Tam, S., et al. (2021). Dual proteome-scale networks reveal cell-specific remodeling of the human interactome. Cell *184*, 3022-3040 e3028. 10.1016/j.cell.2021.04.011.
7. Liberzon, A., Birger, C., Thorvaldsdottir, H., Ghandi, M., Mesirov, J.P., and Tamayo, P. (2015). The Molecular Signatures Database (MSigDB) hallmark gene set collection. Cell Syst *1*, 417-425. 10.1016/j.cels.2015.12.004.
8. Lyu, X., Rowley, M.J., and Corces, V.G. (2018). Architectural Proteins and Pluripotency Factors Cooperate to Orchestrate the Transcriptional Response of hESCs to Temperature Stress. Mol Cell *71*, 940-955 e947. 10.1016/j.molcel.2018.07.012.
9. Vallot, C., Ouimette, J.F., Makhlouf, M., Feraud, O., Pontis, J., Come, J., Martinat, C., Bennaceur-Griscelli, A., Lalande, M., and Rougeulle, C. (2015). Erosion of X Chromosome Inactivation in Human Pluripotent Cells Initiates with XACT Coating and Depends on a Specific Heterochromatin Landscape. Cell Stem Cell *16*, 533-546. 10.1016/j.stem.2015.03.016.
10. Akdemir, K.C., Jain, A.K., Allton, K., Aronow, B., Xu, X., Cooney, A.J., Li, W., and Barton, M.C. (2014). Genome-wide profiling reveals stimulus-specific functions of p53 during differentiation and DNA damage of human embryonic stem cells. Nucleic Acids Res *42*, 205-223. 10.1093/nar/gkt866.
11. Li, Z., Kuppe, C., Ziegler, S., Cheng, M., Kabgani, N., Menzel, S., Zenke, M., Kramann, R., and Costa, I.G. (2021). Chromatin-accessibility estimation from single-cell ATAC-seq data with scOpen. Nat Commun *12*, 6386. 10.1038/s41467-021-26530-2.
12. Neumayr, C., Haberle, V., Serebreni, L., Karner, K., Hendy, O., Boija, A., Henninger, J.E., Li, C.H., Stejskal, K., Lin, G., et al. (2022). Differential cofactor dependencies define distinct types of human enhancers. Nature *606*, 406-413. 10.1038/s41586-022-04779-x.
13. Consortium, E.P., Moore, J.E., Purcaro, M.J., Pratt, H.E., Epstein, C.B., Shoresh, N., Adrian, J., Kawli, T., Davis, C.A., Dobin, A., et al. (2020). Expanded encyclopaedias of DNA elements in the human and mouse genomes. Nature *583*, 699-710. 10.1038/s41586-020-2493-4.
14. Bravo Gonzalez-Blas, C., De Winter, S., Hulselmans, G., Hecker, N., Matetovici, I., Christiaens, V., Poovathingal, S., Wouters, J., Aibar, S., and Aerts, S. (2023). SCENIC+: single-cell multiomic inference of enhancers and gene regulatory networks. Nat Methods *20*, 1355-1367. 10.1038/s41592-023-01938-4.
15. Ishii, S., Kakizuka, T., Park, S.J., Tagawa, A., Sanbo, C., Tanabe, H., Ohkawa, Y., Nakanishi, M., Nakai, K., and Miyanari, Y. (2024). Genome-wide ATAC-see screening identifies TFDP1 as a modulator of global chromatin accessibility. Nat Genet *56*, 473-482. 10.1038/s41588-024-01658-1.
